# Computational Studies of SARS-CoV-2 3CLpro: Insights from MD Simulations

**DOI:** 10.3390/ijms21155346

**Published:** 2020-07-28

**Authors:** Alessandro Grottesi, Neva Bešker, Andrew Emerson, Candida Manelfi, Andrea R. Beccari, Francesco Frigerio, Erik Lindahl, Carmen Cerchia, Carmine Talarico

**Affiliations:** 1 Department HPC, CINECA, via dei Tizii 6, 00185 Roma, Italy; a.grottesi@cineca.it (A.G.); n.besker@cineca.it (N.B.); 2 Department HPC, CINECA, via Magnanelli 6/3, 40033 Casalecchio di Reno (BO), Italy; a.emerson@cineca.it; 3 Dompé Farmaceutici SpA, via Campo di Pile, 67100 L’Aquila, Italy; candida.manelfi@dompe.com (C.M.); andrea.beccari@dompe.com (A.R.B.); 4 Department Physical Chemistry, R&D, Eni SpA, via Maritano 27, 20097 San Donato Milanese (MI), Italy; francesco.frigerio@eni.com; 5 Department of Biochemistry and Biophysics, Science for Life Laboratory, Stockholm University, 17165 Solna, Sweden; erik@kth.se; 6 Department of Pharmacy, University of Napoli “Federico II”, via D. Montesano 49, 80131 Napoli, Italy; carmen.cerchia@unina.it

**Keywords:** SARS-CoV-2, COVID-19, protease, molecular dynamics, 3CLpro

## Abstract

Given the enormous social and health impact of the pandemic triggered by severe acute respiratory syndrome 2 (SARS-CoV-2), the scientific community made a huge effort to provide an immediate response to the challenges posed by Coronavirus disease 2019 (COVID-19). One of the most important proteins of the virus is an enzyme, called 3CLpro or main protease, already identified as an important pharmacological target also in SARS and Middle East respiratory syndrome virus (MERS) viruses. This protein triggers the production of a whole series of enzymes necessary for the virus to carry out its replicating and infectious activities. Therefore, it is crucial to gain a deeper understanding of 3CLpro structure and function in order to effectively target this enzyme. All-atoms molecular dynamics (MD) simulations were performed to examine the different conformational behaviors of the monomeric and dimeric form of SARS-CoV-2 3CLpro apo structure, as revealed by microsecond time scale MD simulations. Our results also shed light on the conformational dynamics of the loop regions at the entry of the catalytic site. Studying, at atomic level, the characteristics of the active site and obtaining information on how the protein can interact with its substrates will allow the design of molecules able to block the enzymatic function crucial for the virus.

## 1. Introduction

A novel coronavirus (severe acute respiratory syndrome coronavirus 2, SARS-CoV-2) has been identified as the pathogen responsible for the outbreak of a severe, rapidly developing pneumonia (Coronavirus disease 2019, COVID-19), which has broken out in Wuhan, China, in 2019. Since then, SARS-CoV-2 has circulated globally, because of its characteristics of strong contagion and high concealment [1]. SARS-CoV-2 is a single-stranded, positive-sense RNA virus belonging to the family of beta-coronavirus. The beta-coronavirus group also comprises the severe acute respiratory syndrome virus (SARS-CoV) and the Middle East respiratory syndrome virus (MERS-CoV). The genome of coronaviruses contains at least six open reading frames (ORFs). The first ORFs (ORF1a/b) directly translate two polyproteins: pp1a and pp1ab. These polyproteins are processed by virally encoded chymotrypsin-like protease (3CLpro) or main protease (Mpro) and one or two papain-like protease (PLpro) into 16 non-structural proteins (nsps). Other ORFs, near the 3’-terminus, encode at least four main structural proteins: spike (S), membrane (M), envelope (E), and nucleocapsid (N) proteins [2]. The spike protein S forms the outer layer of the coronavirus, giving the characteristic crown-like aspect, and initiates host cell invasion via binding to the angiotensin-converting enzyme 2 (ACE2). This process requires S protein priming by host cell serine protease TMPRSS2 [3]. Because there is no human homolog of 3CLpro, and given the critical role 3CLpro plays in the replication of the virus [4], it represents a valid target for the design of antiviral therapies [5,6].

The active 3CLpro is a homodimer, containing two protomers. Each protomer is formed by three domains: six antiparallel β-barrels form domains I and II (residues 8–101 and 102–184, respectively) and host the substrate-binding site, while domain III (residues 201–303) is a globular cluster of five helices that regulates protein dimerization. The 3CLpro possesses a Cys-His dyad, located in the cleft between domains I and II, in which the cysteine thiol functions as the nucleophile in the proteolytic process. The active site is composed of four sites (S1’, S1, S2, and S4).

In the dimer, there exist a number of intermolecular interactions between the two protomers. Specifically, the hydrogen bonds between the N-terminus of a protomer and the domain II of another protomer that helps shaping the S1 pocket of the substrate-binding site; hence, the dimer is the catalytically active form, while the monomer is mostly inactive [7,8]. The dimerization involves other specific intermolecular interactions between two protomers as the salt bridge between N-terminus and domain III of another protomer and the specific electrostatic and hydrophobic interactions between the two different domains III.

The amino acids in substrates from the N terminus to C terminus are numbered as P1–P4 and P1’–P3’; the cleavage site resides between the P1 and P1’, with a Gln residue required in the P1 position of the substrates. The substrate recognition pockets in 3CLpro are named as S1–4, accordingly. The 3CLpro has been the target of extensive efforts in the search for potential drug leads; in this regard, structural biology plays a crucial role, considering the growing number of three-dimensional structures for 3CLpro released so far [8,9,10,11]. For instance, the X-ray crystal structure of the 3CLpro in complex with the inhibitor N3 has been recently released with PDB IDs 6LU7 and 7BQY at 2.16 and 1.7 Å resolution, respectively [9]. Overall, the structure shows a high degree of similarity with that of the SARS-CoV 3CLpro, as expected from the 96% sequence identity [8]. N3 is a time-dependent, irreversible inhibitor of 3CLpro, featuring a vinyl carboxyl ester; this moiety acts as Michael acceptor warhead, trapping the catalytic Cys-145. Notably, two ordered water molecules were observed within the S1 pocket, which is shaped by residues Phe-140, Asn-142, Glu-166, His-163, His-172, and Ser-1 from protomer B [9].

Although the available crystal structures of the 3CLpro provide important insights about atomistic protein–inhibitor interactions, they represent a portion of the possible conformations explored by the enzyme. Therefore, to shed some light on the structural-dynamical behavior of this enzyme, we performed extended all-atoms molecular dynamics (MD) simulations in the microsecond time scale. Such an extended sampling is needed when large amplitude conformational rearrangements have to be addressed [12,13,14,15,16,17]. Moreover, the importance of combining the docking algorithms and virtual screening with dynamic structural information provided by MD simulations, and thus explicitly accounting for the flexibility of both the receptor and the docked ligands, has been widely recognized [18]. To the best of our knowledge, such a long simulation has not been reported for SARS-CoV-2 3CLpro so far. Other computational techniques have been used to access microsecond timescale as aggregate time. An impressive effort is in progress in D. E. Shaw’s group, which is running long simulations on key protein targets of SARS-CoV-2, providing the simulation trajectories to the scientific community for subsequent analysis. 

Herein, we investigate the conformational dynamics of SARS-CoV-2 3CLpro in its monomeric and dimeric forms. Starting from the structure of SARS-CoV-2 3CLpro, we analyze the role played by global and local chain flexibility in the catalytic mechanism. Our results highlight the role played by key residues in the enzyme catalytic pocket, which can be useful for the subsequent phases of drug discovery. In addition, we also find out that the conformational dynamics of loops in the binding pocket entrance are different in the monomeric and dimeric form of the 3CLpro, thus suggesting the importance of the inter-subunits interactions for the catalysis.

## 2. Results

### 2.1. Overall Structure of SARS-CoV-2 3CLpro and MD Setup

**Figure 1:**
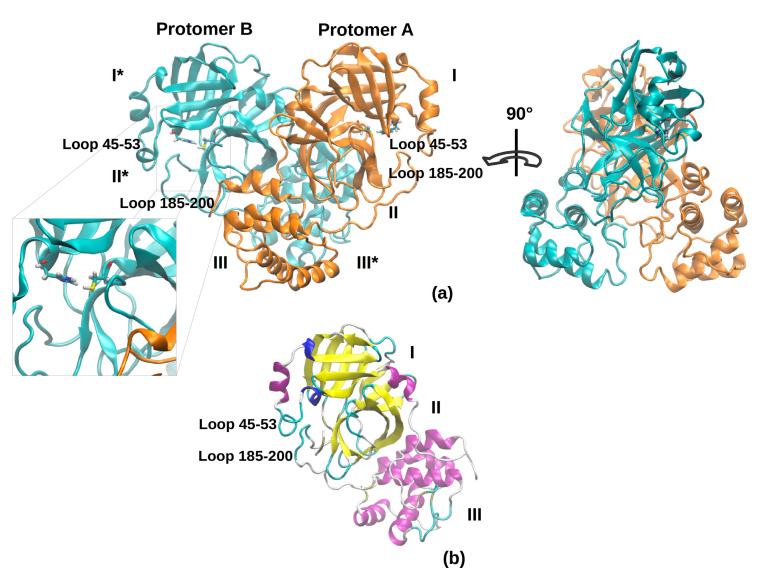
The 3D structure model of 3CLpro in its dimeric (**a**) and monomeric (**b**) form is represented as a cartoon. In the dimeric form (**a**), two protomers are represented in two different colors; domains I, II, and III in chain A, and the corresponding domains I*, II*, and III* in chain B are labeled with roman numbers. In the highlighted box, the catalytic dyad (His-41 and Cys-145) and the range of the residues defining the binding pocket loops are labeled. In the monomeric form (**b**), secondary structure elements are colored in violet (α-Helices), yellow (β-strands), and cyan (loops).

Simulations were started from the X-ray coordinates deposited in the Protein Data Bank (PDB ID: 6LU7) and modified as described in the Methods section. Figure 1 shows the overall topology and conformation of initial SARS-CoV-2 protease in dimeric form. As shown in the figure, 3CLpro is characterized by three main domains. The first domain (I) consists of 103 residues (8–101) and the second one (II) encompasses residues 102–184, and both have an antiparallel β-barrel structure. The third (III) domain (residues 201–303) contains five α-helices and is arranged into a largely antiparallel globular cluster; it is linked to domain II by means of a long loop region (residues 185–200). The protease catalytic site is formed by a Cys-His dyad and the substrate-binding site is located in a pocket between domain I and II [9]. The catalytic pocket is enclosed by two loop regions that physically occlude the path to the catalytic site and are known to play an important role in the catalysis. Independent simulations of the monomeric (protomer A) and dimeric (protomers A and B, denoted chains A and B) forms of SARS-CoV-2 3CLpro have been performed in explicit solvent. As the protease was simulated in the apo form, before starting the simulations, the bound covalent ligand was removed and the side chain of the catalytic Cys-145 was set back to the thiol form. The insights obtained from our simulations provide key reference points for future drug design studies; in fact, the MD trajectories can be deployed to obtain starting structures for a subsequent virtual screening. For this reason, the use of the apo form avoids possible structural bias owing to the presence of a ligand.

### 2.2. Trajectory Stability and Flexibility Analysis

**Figure 2:**
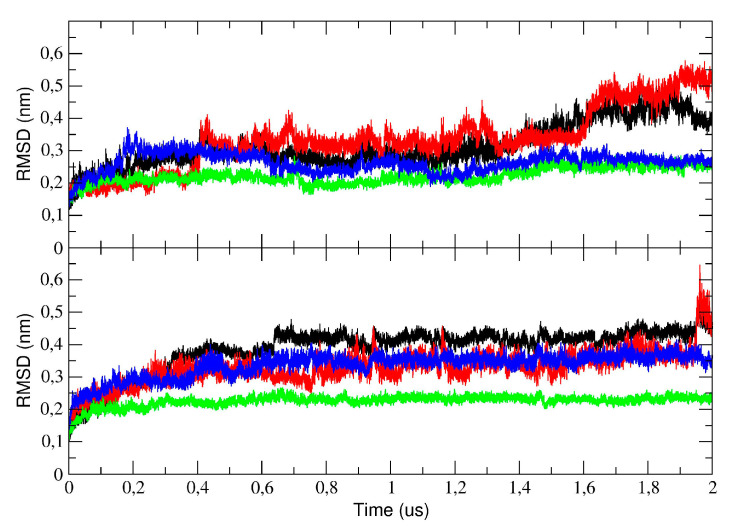
Root mean square deviation (RMSD) of the Cα in the 2 µs molecular dynamics (MD) simulation for the monomer (upper graph) and the dimer (run #1, lower graph). The total RMSD is represented with a black line, while the RMSD of the domain I, II, and III is represented with a red, green, and blue line, respectively.

To sample the structural stability of the 3CLpro during the simulations, we measured the deviation of each structure from the starting crystallographic coordinates after a superposition on the protein Cα atoms. To this end, the root mean square deviation (RMSD) was calculated and is plotted in Figure 2. As the figure shows, the RMSD provides evidence that all the simulated systems have reached convergence of the structural drift by sampling a local potential energy minimum. The figure also shows the RMSD of the individual domains for both the monomer and dimer. In particular, the trajectory range of 0–0.4 µs was sufficient to reach a plateau of the structural drift and provided enough confidence for the convergence of the simulations. Therefore, on the basis of these data, the first 0.4 µs of MD trajectories have been neglected for all subsequent analyses. Besides the simulation of monomeric and dimeric 3CLpro (first run, denoted as run #1), two additional trajectories have been performed of dimeric 3CLpro (hereafter denoted as run #2 and run #3), to improve statistical confidence of the flexibility and PCA analysis. On the basis of the RMSD plots of these additional runs (see Figure S1, Supplementary Materials), for the analysis, we selected the time interval of the trajectories where the conformational drift of all C-alpha atoms was stable.

**Figure 3:**
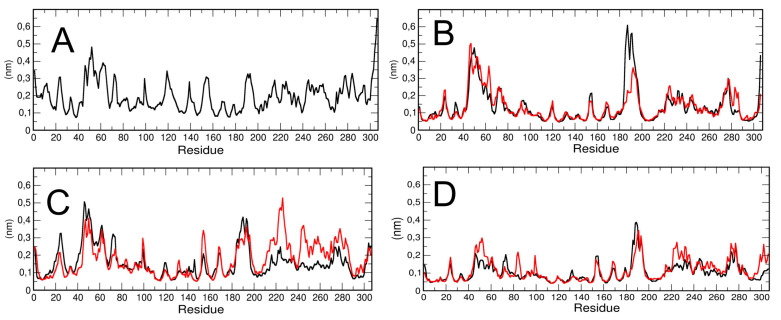
Comparison between the structural flexibility of 3CLpro monomeric and dimeric form. Residue-based root mean square fluctuation (RMSF) of the monomeric 3CLpro Cα (**A**) and the homodimer of run #1 (**B**), run #2 (**C**) and run #3 (**D**) in which different fluctuations of the two protomers are reported (the black curve the chain A (atoms 1–4862) and in red the chain B (atoms 4863–9364).

The residue-based root mean square fluctuation (RMSF) in the trajectory was calculated to measure the flexibility of the residues (Figure 3). Higher RMSF values indicate greater flexibility during the MD simulation. We have computed the RMSF for each chain of the dimeric 3CLpro and for the monomeric simulation (run #1). The results are plotted in Figure 3A,B. The data show that the two chains of dimeric protease have a different RMSF in specific regions of the protein. In particular, chain A, the first part of the long loop region, residues from 180 to 190, connecting domain II and III, has higher flexibility than chain B.

To verify that the computed differences in the RMSF of the two protomers in dimeric 3CLPro were typical, we repeated the calculation on two additional trajectories obtained from different initial velocities starting from the same initial structures (see Methods for the details). The results (see Figure 3C,D) show that, at least in the second trajectory, specific regions of the chain B (from residue 210 to 240 and from residue 241 to 290, encompassing domain III) are more flexible with respect to chain A; in the third trajectory performed, the RMSF analysis shows no significant differences among the two protomers, with the exception of a few residues in domain III. Our findings are also in agreement with a previous study confirming, by a combination of experiments and simulations, that the two protomers in the dimer are asymmetric, and that only one protomer is active at a time [19]. It is worth noting that RMSF in monomeric 3CLpro is higher throughout the polypeptide chain as compared with dimeric 3CLpro RMSF, which is lower, especially in the structured regions. That could reflect protein–protein interactions at the dimer interface that stabilize the overall structure. In addition, the RMSF of the loop region from residue 45 to residue 53 is slightly higher than the corresponding region in the monomer and the long loop region, encompassing residues 183 to 193, is higher in the dimeric than in the monomeric 3CLpro. We speculate a possible correlation of the loop flexibility with interaction of candidate inhibitors.

### 2.3. Essential Dynamics Analysis

All the three MD trajectories of dimeric 3CLpro were analyzed using essential dynamics (see Methods for details) and, in particular, sampled conformational space was analyzed to look for populated conformational sub-states relevant to catalysis. Projection of all trajectories onto a principal plane defined by eigenvectors of the trajectory resulting by concatenating the two chains of the dimer in a single chain trajectory.

**Figure 4:**
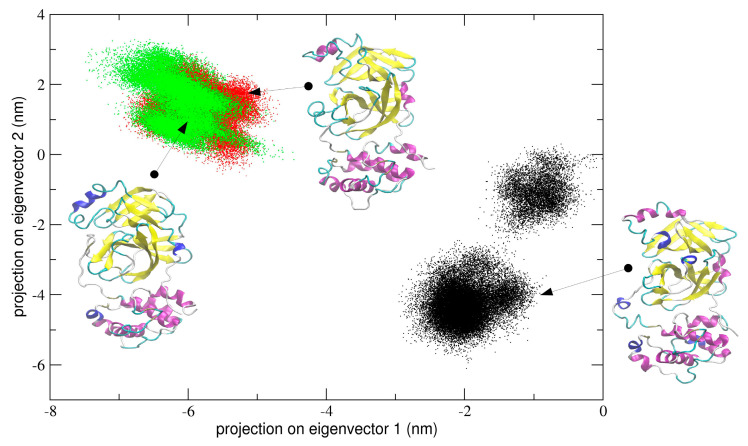
2D projection of the concatenated dimeric protomers trajectory of run #1 on the essential subspace along eigenvectors 1 and 2. The structural basin visited by the chain A (black dots), chain B (red dots) in the dimeric form and the monomeric form (green dots) of 3CLpro is highlighted in different colors. The representative structures of the 2D projection on essential space are reported in the figure with the secondary structure elements colored in violet (α-Helices), yellow (β-strands), and cyan (loops).

In the first simulation, at global level, the conformational dynamics of the dimeric 3CLpro shows that the single chains have different conformational behavior, as evidenced by the projections of Cα coordinated onto the plane defined by the first two eigenvectors (the first principal plane) of the combined trajectory (see Figure 4). As the figure shows, the two chains map on different areas of the principal plane along the first eigenvector (x-axis), suggesting that this collective coordinate discriminates among their internal conformational dynamics. Along the second eigenvector (y-axis), a partial overlap of the two chains is observed, thus evidencing that, along with this component, the two chains partially sample the same subspace region. The first two eigenvectors of Figure 4 account for 80% of all cumulative fluctuations (54% and 26% for the first and second eigenvector, respectively). We have projected the monomeric MD trajectory onto this essential plane. Interestingly, the area where the monomer projects (green dots in Figure 4) onto this plane is highly overlapped with the corresponding area of the chain B of 3CLpro, visiting the same conformational region. At the molecular level, the structural elements that account for these differences in the first principal plane of Figure 4 are located mainly on domain I and in particular on the loop regions of the two chains of the 3CLpro dimer.

The same analysis performed on the second and third trajectory provided similar results in terms of conformational dynamics defined by a principal plane. In particular, for run #2 and #3, the corresponding sampled spaces are reported in Figure S2 (Supplementary Materials). As the figure shows, and similarly to what was observed for run #1, the two chains sample distinct regions of the principal plane, where a clear split of the sampled spaces of chain A and B in dimeric 3CLpro is shown.

Interestingly, the projection of the monomeric trajectory onto the first principal plane of run #2 and #3 showed that, in run #2 and #3, the first eigenvector of the concatenated trajectory partially overlaps with that of chain A in run #2 and with both projections of chain A and B in run #3; in the second eigenvector, the monomeric projection overlaps with both chain A and B of both run #2 and #3, suggesting that local internal dynamics of all chains (monomer and protomerA and protomerB) are similar.

In order to quantitatively compare the similarity between different principal modes, the inner product of the first eigenvector (which accounts for about 76–83% of all essential motions) of the corresponding protomers has been calculated. The inner product between chain A and B is 0.21 and the corresponding value for chain A and the monomer is 0.17, suggesting that the first essential mode in these two cases is different. The inner product between the first eigenvectors of chain B and the monomer is 0.82, thus indicating a high similarity of this essential mode. This quantitative analysis suggests that chain B in dimeric 3CLpro is more similar to monomeric protease as compared with chain A.

**Figure 5:**
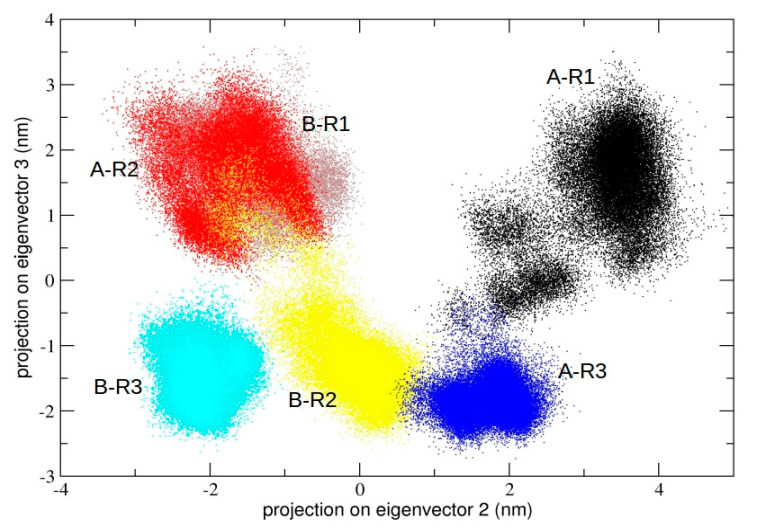
2D projection of all trajectories onto the principal plane defined by the second and third eigenvector of the concatenated trajectory of all three dimeric 3CLpro runs (run #1, run #2, and run #3). The projections of the chains from the three independent simulations are shown with different colors according to the following scheme: A-RX and B-RX (with X = 1,2,3) for chain A and B for run #1, #2, and #3, respectively.

We also calculated the principal components defined by the concatenated trajectories of all the chains of 3CLpro of the three independent simulations, resulting in a total of 10 µs. The rationale was to define a common principal plane able to discriminate globally and locally among the dynamics of individual chains. It is worth noting (see Figure 5) that the second principal plane (defined by the second and third eigenvector) of this longer concatenated trajectory discriminates between protomeric chains of all trajectories. At the molecular level, the structural elements that mainly account for the detected differences correspond to the loop region from residue 45 to 53 and from residue 183 to 193, which are located at the entrance of the 3CLpro binding pocket (see Figure S3, Supplementary Materials). In particular, the plot shows that each individual protomer within a single dimeric trajectory maps onto distinct regions of the conformational space defined by the plane.

**Figure 6:**
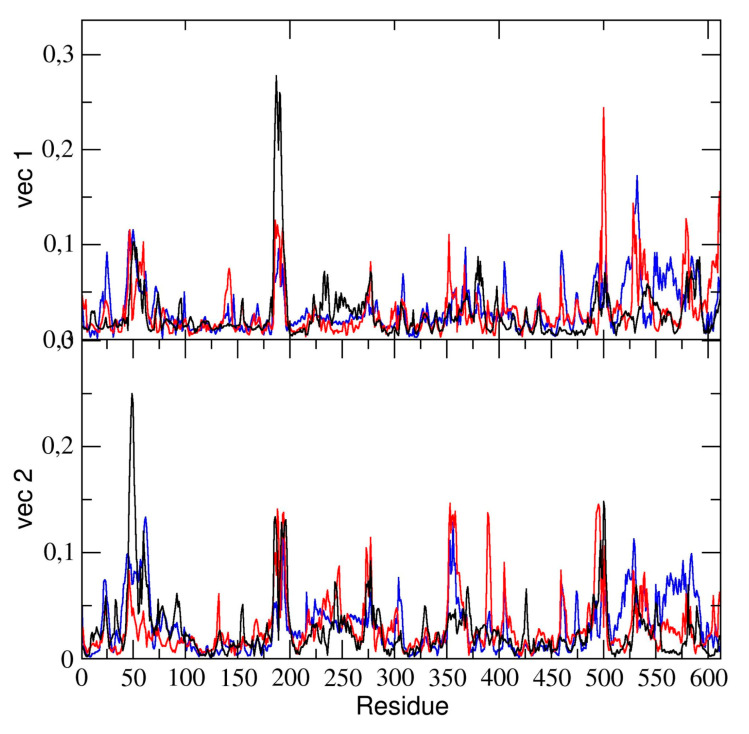
Absolute components of the first two C-α eigenvectors for the dimeric 3CLpro for runs #1 (black line), #2 (blue line), and #3 (red line).

The essential dynamics analysis of the dimeric 3CLpro trajectory was also performed. The residue components of these eigenvectors provide useful information on the contribution of specific residues to the overall global dynamics. To this end, the positional fluctuations components of the first and second eigenvectors in dimeric 3CLpro are shown in Figure 6. Clearly, the figure shows that some regions of the protein structure have a higher contribution in the eigenvectors components, thus providing evidence that those regions describe the essential motion of the 3CLpro dimer. It is worth noting that the contribution of the two chains is different. In particular, residues of chain A contribute significantly to the first (principal) eigenvector (residues 50–51, 187–189). These same residues of chain A also contribute to the second eigenvector. Residues 189–191 of chain B also have a higher component of the second eigenvector. This was also observed in the flexibility analysis by RMSF calculations (vide supra).

We compared the residue components of all dimeric 3CLpro trajectories and found (see Figure 6) that, along the first and second eigenvector, the main contributions to flexibility along those components is determined by the same residue ranges, thus providing evidence that, globally, the 3CLpro dynamics are the same in all three trajectories.

### 2.4. Dynamic Behavior of the Binding Pocket

**Figure 7:**
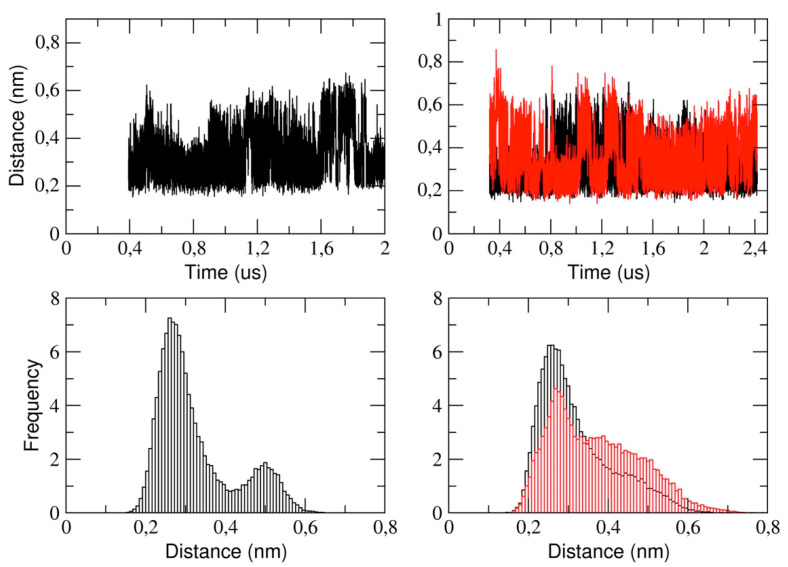
The distance computed between the catalytic dyad residues His-41 and Cys-145 during MD simulations for the monomeric (upper left graph) and for the dimeric (upper right graph) form of 3CLpro. In the lower graph, the frequency of the distances is represented for the monomer on the left and for the dimer on the right. For the dimer, the two chains are highlighted in different colors: chain A in black line and chain B in red line in both graphs.

This section reports the analysis of the binding pocket regions of 3CLpro. We determined the structural stability of the pocket region by measuring the distance between the catalytic dyad residues, that is, His-41 and Cys-145 (see Figure 7). The figure shows that, in the monomeric form of the protease, the His-41 and Cys-145 distance is distributed such that we observe two distinct sub-states characterized by a mode value of 0.26 nm and 0.5 nm, respectively. In the dimeric 3CLpro, the minimum inter-residue distance in chain A is 0.26 nm. This distance is comparable to the starting distance in the X-ray structure (0.19 nm in chain A and 0.26 nm in chain B). In chain B, we observe a similar value of the mode, (0.27 nm) with a second spread peak at a larger distance that is similar to what observed in the corresponding analysis of the monomer.

This also indicates that, in chain A, the two catalytic residues remain at a short distance throughout the simulation, while they fluctuate in chain B. This is also evident analyzing the corresponding histograms of the distance (Figure 7, lower panels). It is worth noting that the behavior of the corresponding distance in the monomer is similar as in chain B of dimeric protease.

**Figure 8:**
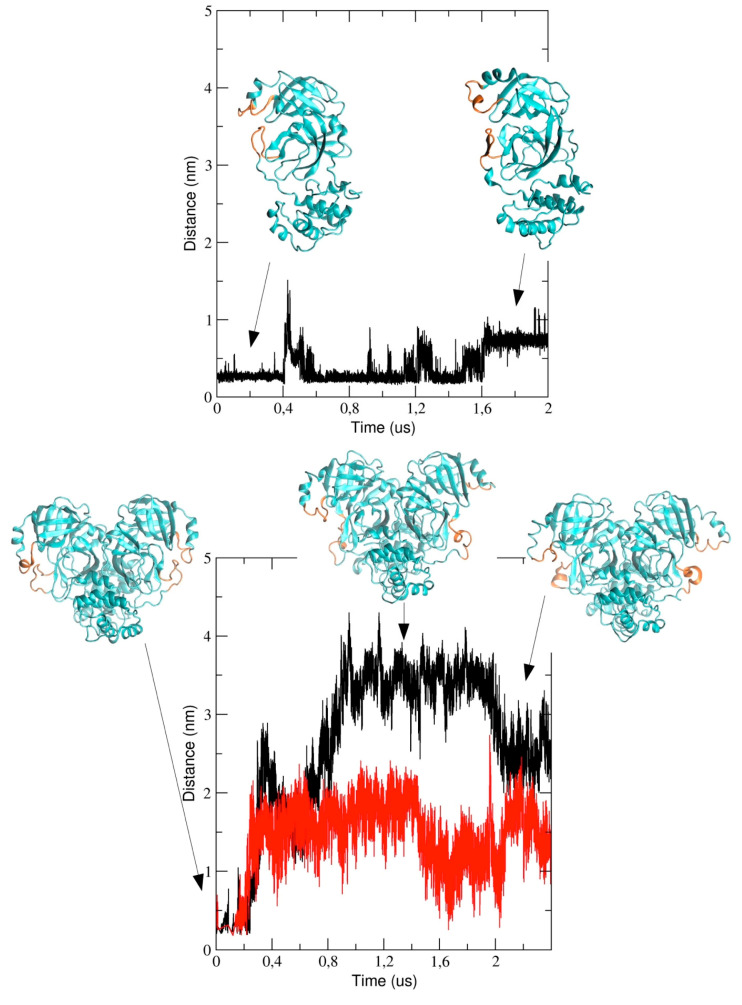
The distance computed between Met-49 and Arg-188 as the description of the two loop distance in the MD simulations for the monomeric 3CLpro (in the upper graph) and for the two chains in the dimeric 3CLpro (black line for chain A and red line for chain B in the lower graph) Representative structures, collected at different timesteps (indicated by black arrows) for the monomeric and dimeric 3CLpro are shown in cyan, with the two loops highlighted in orange.

We also analyzed the conformational dynamics of the loops encompassing residues 44 to 53 and 184 to 193, which are located at the entrance of the pathway to the binding pocket. In particular, we measured the inter-residues distance between Met-49 and Arg-188 (see Figure 8). The figure shows that the distance increases in the course of the simulation and this implies a structural re-arrangement of the two loops, thus providing evidence that the binding pocket region becomes more exposed to the bulk solvent. It is worth noting that the two loops behave differently in the two chains of dimeric 3CLpro; in particular, the distance is larger in chain A (black graph in Figure 8) as compared with chain B and, after 2 μs, the inter-loop distance in the two chains becomes comparable.

Similar results were obtained for runs #2 and #3 (see Figure S4, Supplementary Materials). In run #2, the loops dynamics is more dampened and the minimum distance between Met-49 and Arg-188 is below 2 nm throughout the trajectory for both chain A and B (with only a few exception at about 0.5 μs and 1.5 μs). In run #3, the loop dynamics is characterized by a reduced mobility (similarly to run #2), although a clear difference in the inter-loop distance is evident, in particular after 1 μs. The main outcome of this analysis provides evidence that, in all three simulations of dimeric 3CLpro, the interloop distance of chain A and B are not fully correlated, thus suggesting that the loops dynamics in the two protomers is independent from each other. This further support the hypothesis that chain A and B in 3CLpro dimer are different from a dynamical viewpoint (see Discussion).

**Figure 9:**
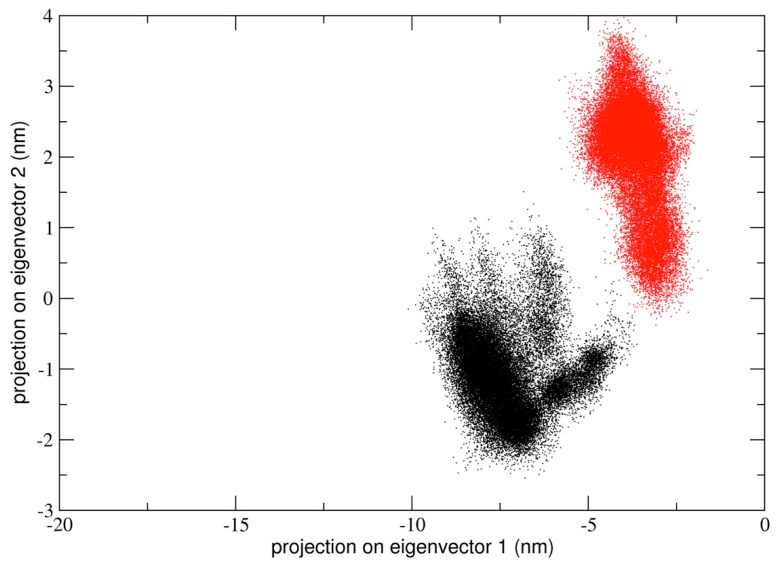
2D projection of the binding pocket region trajectory in the dimeric form of the 3CLpro on the essential subspace along eigenvectors 1 and 2. The structural basin visited by chain A (black dots) and chain B (red dots) is highlighted in different colors.

To characterize in more detail the role played by residue flexibility in the binding pocket, we performed the essential dynamics analysis of the backbone atoms of the subset of residues in the binding pocket, and the results are plotted in Figure 9.

The two chains sample differently the essential space of the binding pocket. Although a small degree of overlapping is present along both the first and second eigenvector, these two components (which account for 93% of all fluctuations of the binding pockets backbone atoms, 85.6% and 7.5% for the first and second eigenvector, respectively) discriminate the behavior of the two sampled spaces, thus suggesting that, in the apo state, the two pockets are different from a dynamical viewpoint.

**Figure 10:**
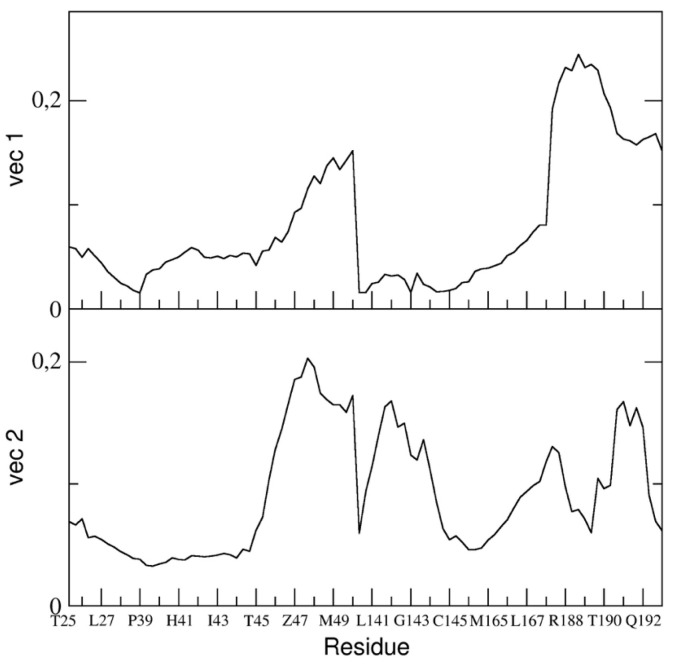
Absolute components of the first two backbone eigenvectors for the residues describing the binding pocket in the dimeric form of the 3CLpro.

In particular, we reported the eigenvector components of the binding pocket residues of the first and the second eigenvectors in Figure 10.

The figure shows that the fluctuation of the backbone atoms of residues of the two loops, residue range 45–49 and 187–192, are correlated and contribute to a high extent to the main dominant motions.

**Figure 11:**
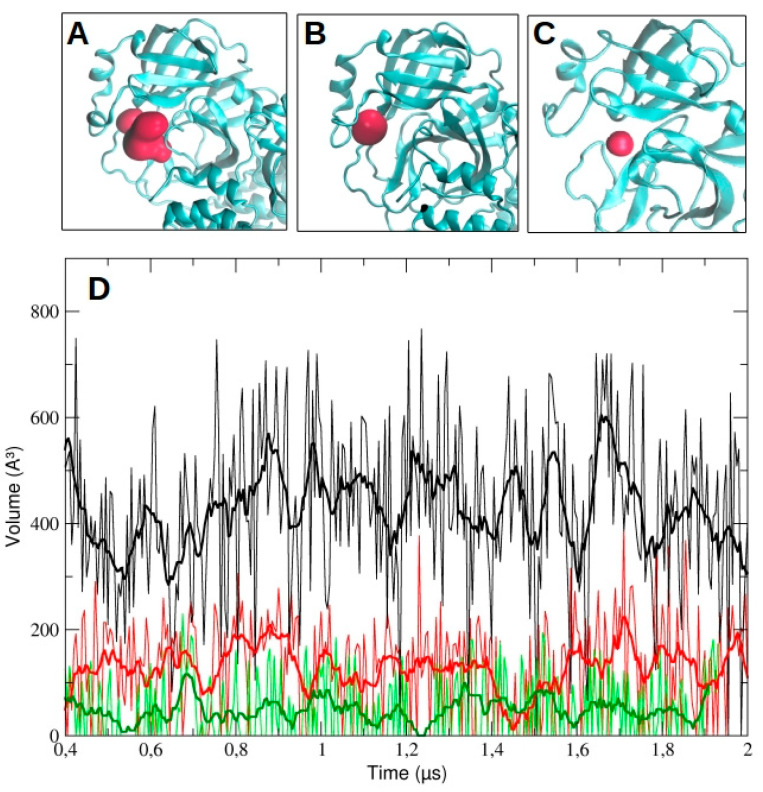
Pocket detection and evolution during MD simulation trajectory. The binding pocket volume was identified by MDpocket and later monitored for changes in volume. The pocket volume is represented in red surface in the dimeric 3CLpro ((**A**) chain A, (**B**) chain B) and in monomeric form (**C**), represented in cyan. In (**D**), the comparison of the evolution of the volume pocket in chain A (black line) and chain B (red line) of the dimer and of the monomer (green line) is represented. The bold line is a smoothed curve of the respective changes in volume line, intended to clarify the behavior of the pocket.

We then analyzed the evolution of the binding pocket volume, as determined by MDpocket analysis (see Methods for details). In particular, we measured the volume as a function of time for chain A and B in dimeric 3CLpro and for the monomeric protease. The data are reported in Figure 11 for run #1, run #2, and run #3 (Figure S5, Supplementary Materials). As the figure shows, the volume in chain A is higher than in chain B throughout the whole simulation time (428.5 ± 154.1 A^3^ and 120.2 ± 93.6 A^3^, respectively). For comparison, the average volume for the monomeric pocket is 49.6 ± 61.4 A^3^. On the contrary, in both runs #2 and #3, we observed a larger accessible volume in chain B than in chain A (Figure S5, Supplementary Materials). In particular, in run #2, the average volume was 500.1 ± 164.9 A^3^ and 305.2 ± 163.3 A^3^ for chain B and A, respectively; in run #3, the average volume was 952.1 ± 227.4 A^3^ and 123.0 ± 112.1 A^3^ for chain B and A, respectively.

The large differences observed among the pocket volumes are mainly ascribable to the conformational rearrangements of the two loops that regulate the accessibility to the catalytic site. This is clearly evident in run #2, where the loop 187–192 moves backward, opening up a significant volume (Figure S5, Supplementary Materials). On the other hand, the dramatic increase in the pocket volume of the protomer B in run #3 is mainly the result of a significant conformational rearrangement of the domains I and II and their respective secondary structure elements, as well as domain III of the protomer A.

## 3. Discussion

The computational study presented here reports the analysis of 2 µs trajectories of the monomeric and the dimeric form of the SARS-CoV-2 3CLpro. It is worth noting that the starting structure of dimeric 3CLpro of our simulation was taken from the holo 3CLpro where the ligand was removed. This could potentially affect the conformational space accessible to our simulated protein throughout the simulation. Notwithstanding, our data provide evidence that the conformational dynamics of the protein chain is different in the dimeric 3CLpro as compared with the monomeric form. In particular, the flexibility of the protein chain is higher in the monomeric 3CLpro than in the dimer and the long loops connecting domain II and III account for most of the flexibility of the protease. The loops regions provide access to the binding site and the catalytic dyad and, therefore, play an important role in the catalytic activity. This is evidenced when the minimum inter-residues distance between Met-49 and Arg-188, which increases during the simulation, is computed. Interestingly, in run #1, the distance increase is more evident in chain A than in chain B, even though such a difference is less obvious at the end of the simulation (see Figure 8). These data also provide evidence that the loop regions account for the largest motions present in the structure, as also evidenced by principal component analysis (see Figure 10). On the other side, the Cys-145-His-41 distance as a function of simulation time was monitored during the MD calculation, and was found to fluctuate in the range of 0.26 nm and 0.5 nm for the monomer, whereas it is roughly constant at a value of 0.26 nm in chain A of the dimer. Instead, in chain B, a trend similar to the monomeric form was observed. These findings suggest that, in chain A of the dimer, the catalytic dyad remains within an H-bond distance, while in the monomer and in chain B of the dimer, there is likely a disruption of the interaction between the two entities. Therefore, it is tempting to speculate that the catalytic residues are able to also modulate the opening of the active site and substrate binding in a concerted manner. The longer distances between the residues of the catalytic dyad likely result in the inactivation of the monomer, whereas only chain A in the dimer would be catalytically competent.

One can also speculate that the dynamical behaviour of dimeric 3CLpro may reflect an overall mechanism of catalysis according to an induced fit model (as was determined for SARS-CoV) [20]. The principal components analysis performed on the concatenated trajectory of dimeric 3CLpro seems to suggest so, as we found that the two chains of dimeric 3CLpro map on different regions of the conformational space defined by the first two eigenvectors (see Figure 4 and Figure S2), thus providing evidence that they are dynamically different and only when a specific interaction with a ligand is achieved, the two chains converge to a similar structural arrangement, experimentally observed in the crystal structures released so far [8,9]. It is worth noting that the loop regions account for most of the global motion of the dimeric 3CLpro (see Figure 6, Figure 10 and Figure S3).

Further evidence that the two chains are dynamically different and become active according to an induced fit mechanism is provided by comparing the volumes of the binding pocket as a function of simulation time (Figure 11 and Figure S5). The overall results showed that, when there is an increase of the binding pocket average volume in one chain, a decrease occurs in the other chain of the dimer.

A further conclusion that can be drawn is that the intermolecular interactions between the two chains in the dimer, in particular the interactions between the N-terminus and domain III of one monomer, are in turn critical to stabilize the residues of the catalytic pocket in the active form, thus ensuring the successful proceeding of the catalytic cycle in the dimer. This further supports that dimerization is important for the enzyme activity. Our findings are in agreement with previous studies regarding the 3CLpro of SARS-CoV, showing that the right conformation for catalysis in one protomer can be induced upon dimer formation and that the enzyme may follow the association, activation, catalysis, and dissociation mechanism for activity control [19].

Our analysis, carried out on a microsecond time scale, highlighted the higher level of complexity of the dimeric form dynamics. Indeed, it is also possible that, in the dimer, one protomer is active while the other one is basically inactive, or even that the two protomers may exchange conformation. 

Overall, this study provides useful molecular insights into the dynamics and mechanism of the functional conformation changes of SARS-CoV-2 3CLpro, which may be of great interest in the search for more effective inhibitors.

## 4. Materials and Methods

The initial structure for monomeric 3CLpro and its dimeric form (Biological Assembly) was taken from the PDB web site (PDB ID: 6LU7, the first X-ray structure of 3CLPro available when we started this study). Both proteases were simulated in the apo form and, therefore, covalently bound ligand in the initial PDB structure was removed and the cysteine residue was set in its reduced state. Tautomeric and protonation states for all titratable residues were set at their default state at pH = 7.

The total number of atoms (protein + solvent + counterions) in the monomer and dimeric 3CLpro was 62,775 and 69,570, respectively.

MD simulations were performed with GROMACS 2020.2 (www.gromacs.org) with the AMBER99 force field [21,22,23]. All simulated structures were centered typically in a triclinic or dodecahedron box with a minimum distance of 1.0 nm between each atom of the protein and the box to reduce and the TIP3P water model was used to solvate the system [24]. The ionic strength was adjusted to make sure all simulations were electrically neutral. Energy minimization was executed by the steepest descent method and the conjugated gradient method for the subsequent 50,000 steps. Nonbonded forces were modeled using the particle-mesh Ewald (PME) method with a cutoff distance of 10 Å, used for all simulations. A stepwise procedure was used to equilibrate the system and consisted of a first cycle of 100 ps positional restraints MD with force constant of 1000 kJ mol-1 nm-2 and applied to the atoms of the protein with solvent atoms free to move, followed by 100 ps MD simulations in the Isothermal–Isobaric (NPT) ensemble to equilibrate pressure and temperature. The initial velocities were taken randomly from a Maxwellian distribution at 300 K. The temperature was held constant (300) K using the V-rescale algorithm. Pressure was determined using the Parrinello–Ramhan barostat [25]. Long-range electrostatic interactions were calculated using the particle mesh Ewald summation methods [26]. Lennard–Jones interactions were calculated using a cutoff of 1 Å. The pair lists were updated every 400 steps. The LINCS algorithm [27] was used to constrain h-bonds. The time step was 2 fs and simulations were 2–3 µs long, typically, and coordinates were saved every 50.0 ps. Three independent simulations with different initial velocities were performed for dimeric 3CLpro. To study the larger amplitude protein motions (also called collective motions or large-scale concerted motions), the essential dynamics analysis (also known as the principal component analysis (PCA)) was done [28]. Briefly, the covariance matrix of the atomic positions is built from the MD simulations on a selected group of atoms (usually Cα). From the diagonalization of such a matrix, a set of eigenvectors and associated eigenvalues is obtained. The eigenvectors represent the principal motion directions of the system and, therefore, they are used to describe the “essential” protein modes, which often represent the functional ones. In this way, the fastest motions present in the simulations, which describe biologically not relevant motions (i.e., vibrations), are excluded, making it possible to represent the protein dynamics in a reduced space, as defined by the eigenvectors, which approximates well the overall molecular motions. The covariance matrices, using the GROMACS g_covar tool, were constructed from the Cα atoms of proteins of the MD trajectory of the 3CLpro both in dimeric form and on concatenated trajectory of individual protease chains to compare the different simulations (the concatenated trajectories are defined either from chain A and chain B within the same run or chain A and chain B of all three independent simulations, run #1, run #2, and run #3. See the results section for details). The GROMACS gmx anaeig tool was used to calculate the 2D projections with respect to the first two eigenvectors and eigenvector components to the selected eigenvectors.

The distance between the two residues in the catalytic dyad and between two residues in two loops adjacent the binding pocket was calculated as a minimum distance between the two selected residues with the gmx mindist tool in GROMACS.

For the description of the binding pocket, residues within a distance of 0.4 nm from the following co-crystallized inhibitors were selected: N3, PDB ID 6LU7 [9]; baicalein, PDB ID 6M2N [11]; 13b, PDB ID 6Y2F [8].

This selection provided 31 residues, including the catalytic dyad, and portion of the long loop connecting domain II and domain III. The residues are from 25 to 28, 39 to 49, 140 to 145, 164 to 167, and 189 to 192.

Detection and evolution of the active pocket volume during the molecular dynamics simulations were carried out with MDpocket software (http://fpocket.sourceforge.net) [29]. Pocket detection was performed automatically by MDpocket, based on volume density detection. Then, we selected the grid points corresponding to the binding pocket region. Pocket volume calculations of the MD simulations were done every 100 frames in the equilibrated trajectories after fitting to the initial energy-minimized structure.

As a part of our study, all the trajectories of the 3CLpro monomer and dimer are made publicly available at the following url: www.exscalate4cov.network.

## Supplementary Materials

Supplementary materials can be found at https://www.mdpi.com/1422-0067/21/15/5346/s1.

## Abbreviations

SARS-CoV-2	Severe acute respiratory syndrome 2
COVID-19	Coronavirus disease 2019
SARS-CoV	Severe acute respiratory syndrome virus
MERS-CoV	Middle East respiratory syndrome virus
ORF	Open reading frames
3CLpro	Chymotrypsin-like protease
Mpro	Main protease
PLpro	Papain-like protease
nsp	Non-structural proteins
ACE2	Angiotensin-converting enzyme 2
MD	Molecular dynamics
PDB	Protein Data Bank
RMSD	Root mean square deviation
RMSF	Root mean square fluctuation
PCA	Principal components analysis
ED	Essential dynamics
