# Supplementary material for: Computational Studies of SARS-CoV-2 3CLpro: Insights from MD Simulations
Source: Int J Mol Sci. 2020 Jul 28;21(15):5346. doi: 10.3390/ijms21155346 (PMC7432786; doi:10.3390/ijms21155346)

**Computational Studies of SARS-CoV-2 3CLpro: Insights from MD Simulations**

**Alessandro Grottesi ^1#^, Neva Bešker ^1#^, Andrew Emerson ^2^, Candida Manelfi ^3^, Andrea R. Beccari ^3^, Francesco Frigerio ^4^, Erik Lindahl ^5^, Carmen Cerchia ^6^ and Carmine Talarico ^3^***

^1^ CINECA HPC Department, via dei Tizii 6, 00185, Roma, Italy; [a.grottesi@cineca.it](mailto:a.grottesi@cineca.it) (A.G.); [n.besker@cineca.it](mailto:n.besker@cineca.it) (N.B.)

^2^ CINECA HPC Department, via Magnanelli 6/3, 40033, Casalecchio di Reno (BO), Italy; [a.emerson@cineca.it](mailto:a.emerson@cineca.it) (A.E.)

^3^ Dompé Farmaceutici SpA, via Campo di Pile, 67100, L’Aquila, Italy; candida.manelfi@dompe.com (C.M.); andrea.beccari@dompe.com (A.R.B.); [carmine.talarico@dompe.com](mailto:carmine.talarico@dompe.com) (C.T.)

^4^ Eni SpA, R&D, Physical Chemistry Department, via Maritano 27, 20097, San Donato Milanese (MI) Italy; [francesco.frigerio@eni.com](mailto:francesco.frigerio@eni.com) (F.F.)

^5^ Swedish e-Science Research Center, KTH Royal Institute of Technology, Scilife Lab Tomtebodavägen 23a, Stockholm, Sweden; [erik@kth.se](mailto:erik@kth.se) (E.L.)

^6^ Department of Pharmacy, University of Napoli “Federico II”, via D. Montesano 49, 80131, Napoli, Italy; carmen.cerchia@unina.it (C.C.)

***** Correspondence: [carmine.talarico@dompe.com](mailto:carmine.talarico@dompe.com); Tel.:+39 3483796369 (C.T.);

^#^ These authors contributed equally to this work.

**Figure S1**. Root mean square deviation (RMSD) of the Cα for runs #2 (upper graph) and #3 (lower graph). The total RMSD is represented with a black line, while the RMSD of the domain I, II and III is represented with a red, green and blue line, respectively.


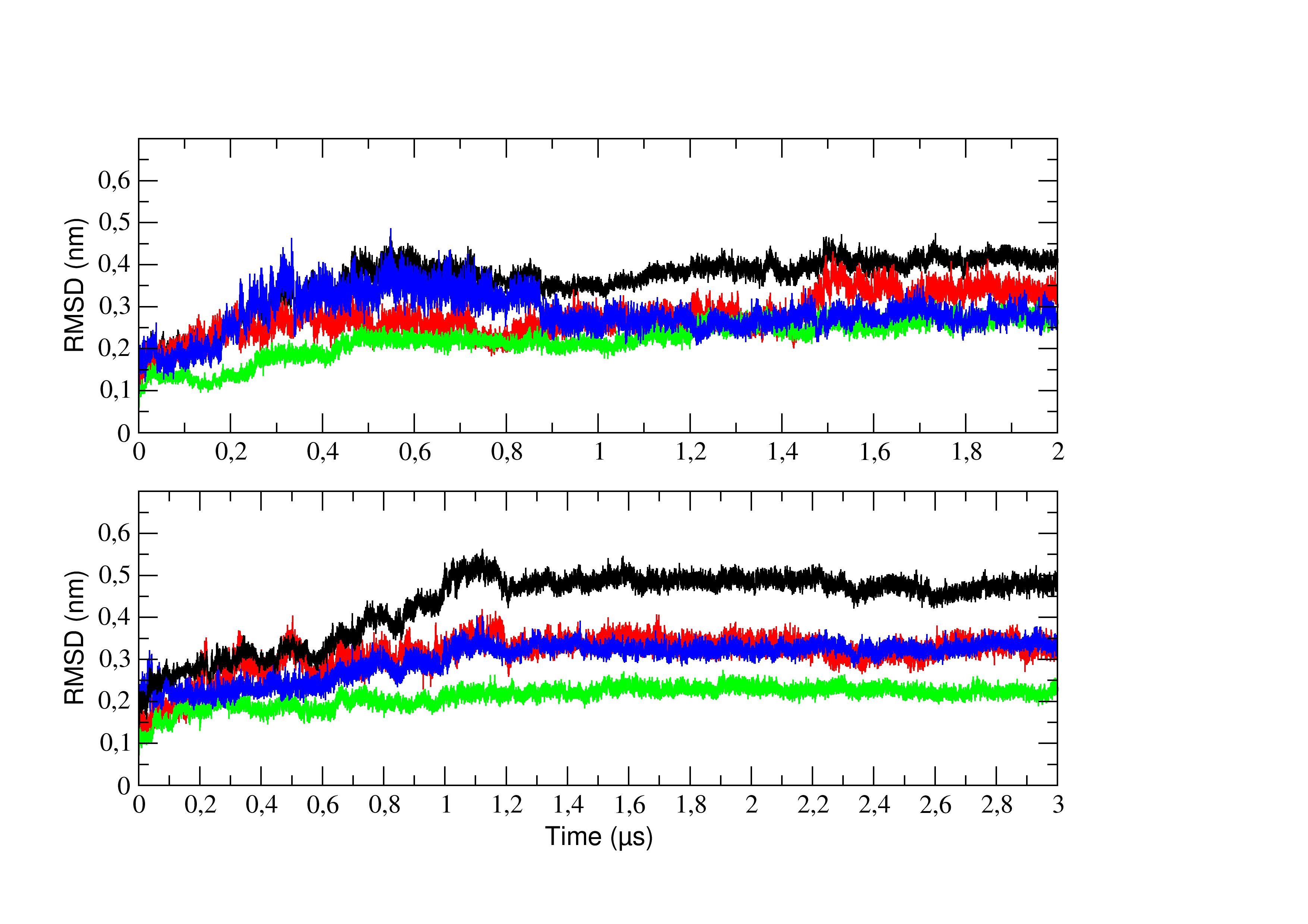


**Figure S2**. 2D projection of the concatenated dimeric protomers trajectory on the essential subspace along eigenvectors 1 and 2 for run #2 (upper panel) and #3 (lower panel). The structural basin visited by chain A (black dots), the chain B (red dots) in the dimeric form of 3CLpro is highlighted in different colors. The projection of the monomeric trajectory is also shown (green dots). The representative structures of the 2D projection on essential space are reported in the figure with the secondary structure elements colored in violet (α‐Helices), yellow (β‐strands), and cyan (loops).


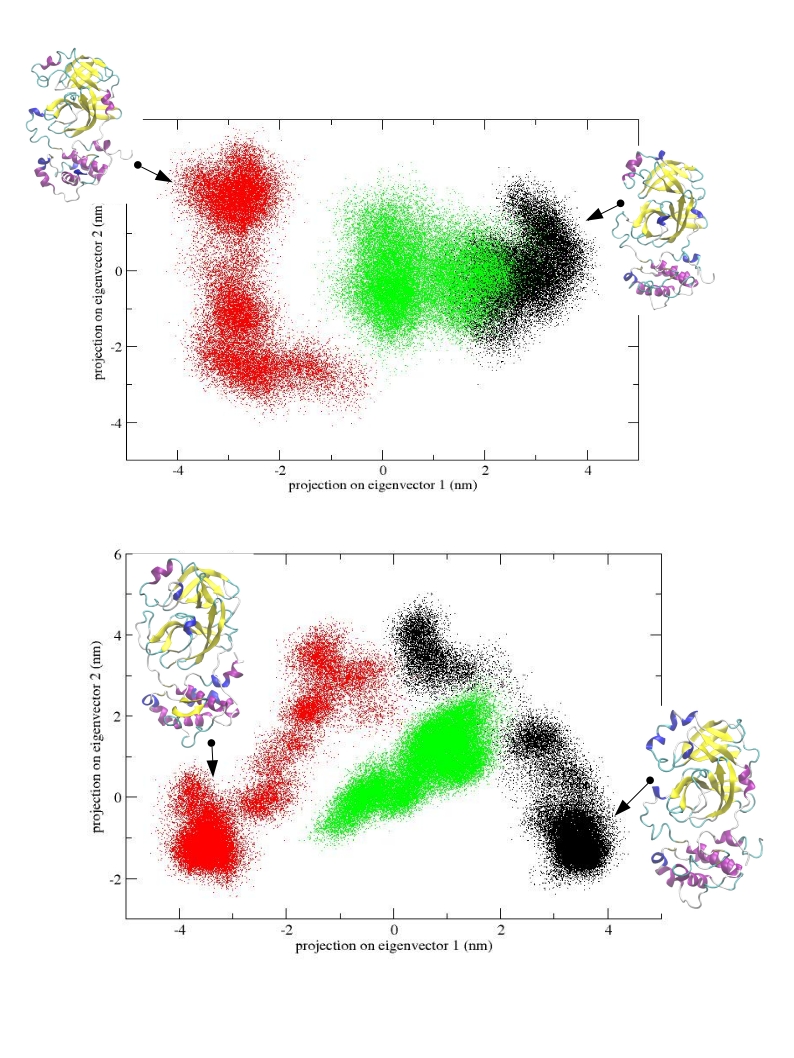


**Figure S3.** Absolute components of the first three C-a eigenvectors obtained by concatenating all trajectories of all protomers from each independent 3Clpro dimeric runs.


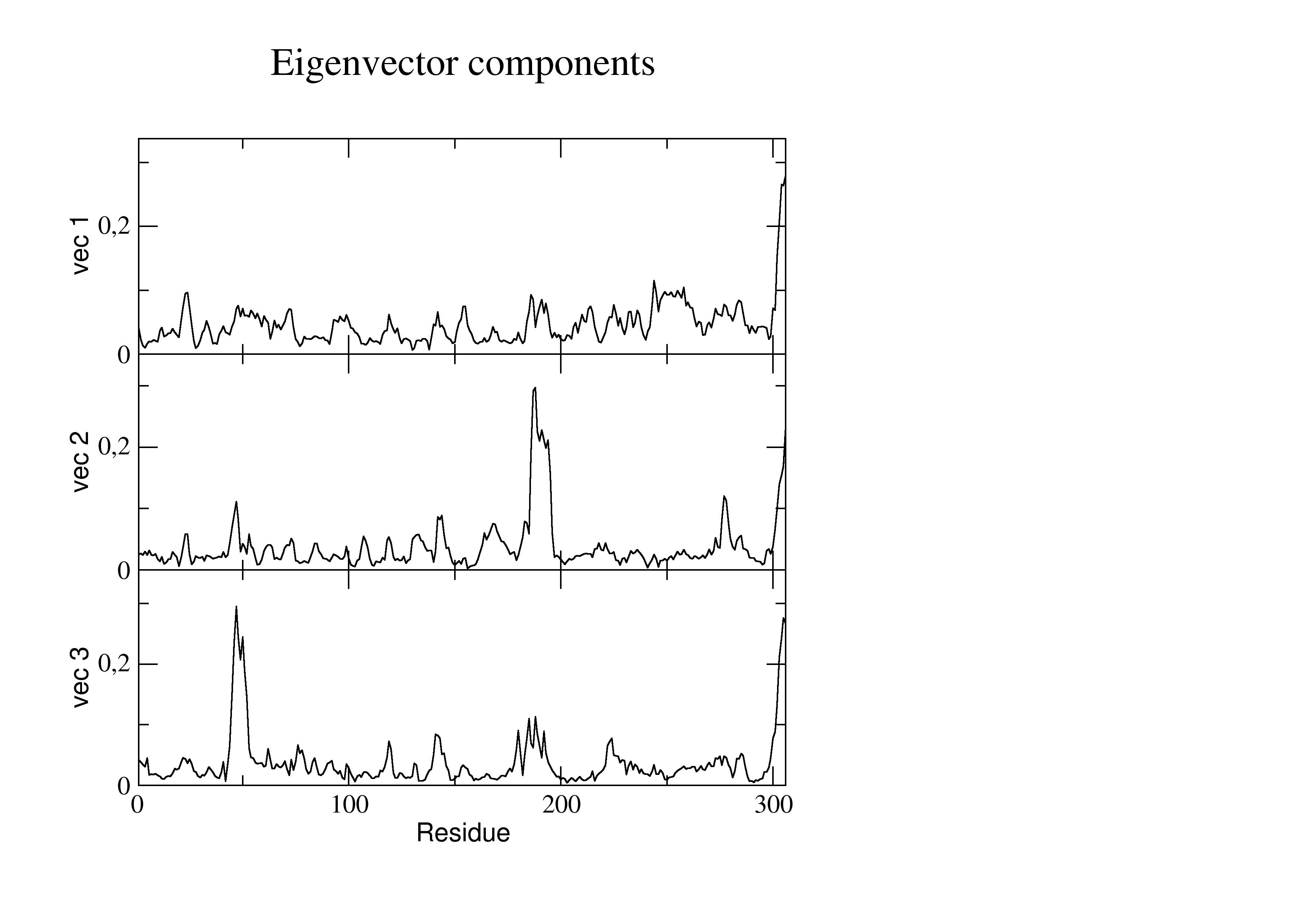


**Figure S4**. The distance computed between Met-49 and Arg-188 as the description of the two loop distance in the MD simulations for the two chains of the dimeric 3CLpro (black line for the chain A and red line for the chain B) in run #2 (upper graph) and #3 (lower graph).


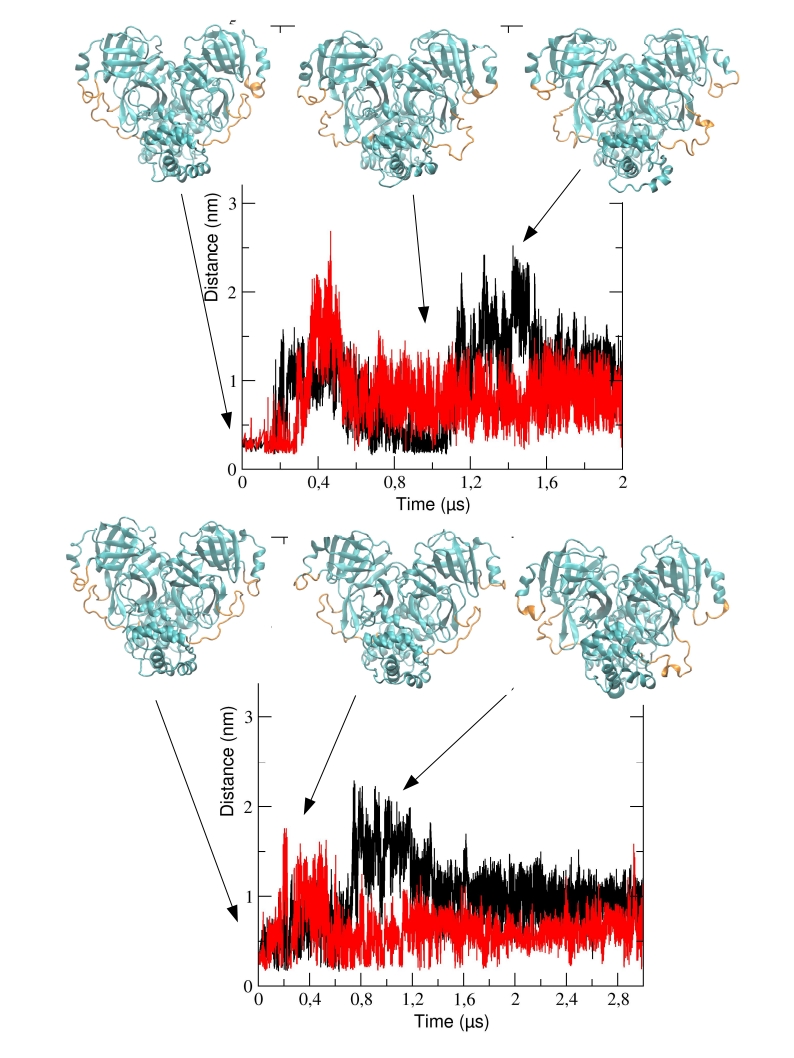


**Figure S5**. Pocket detection and evolution during MD simulation trajectory. The binding pocket volume was identified by MDpocket and later monitored for changes in volume. The plot shows the comparison of the evolution of the volume pocket in chain A (black line), chain B (red line) of the dimeric 3CLpro in run #2 (upper graph) and #3 (lower graph). The bold line is a smoothed curve of the respective changes in volume line, intended to clarify the behavior of the pocket. The pocket is represented as a red surface in the dimeric 3CLpro ((A) the chain A, (B) chain B).


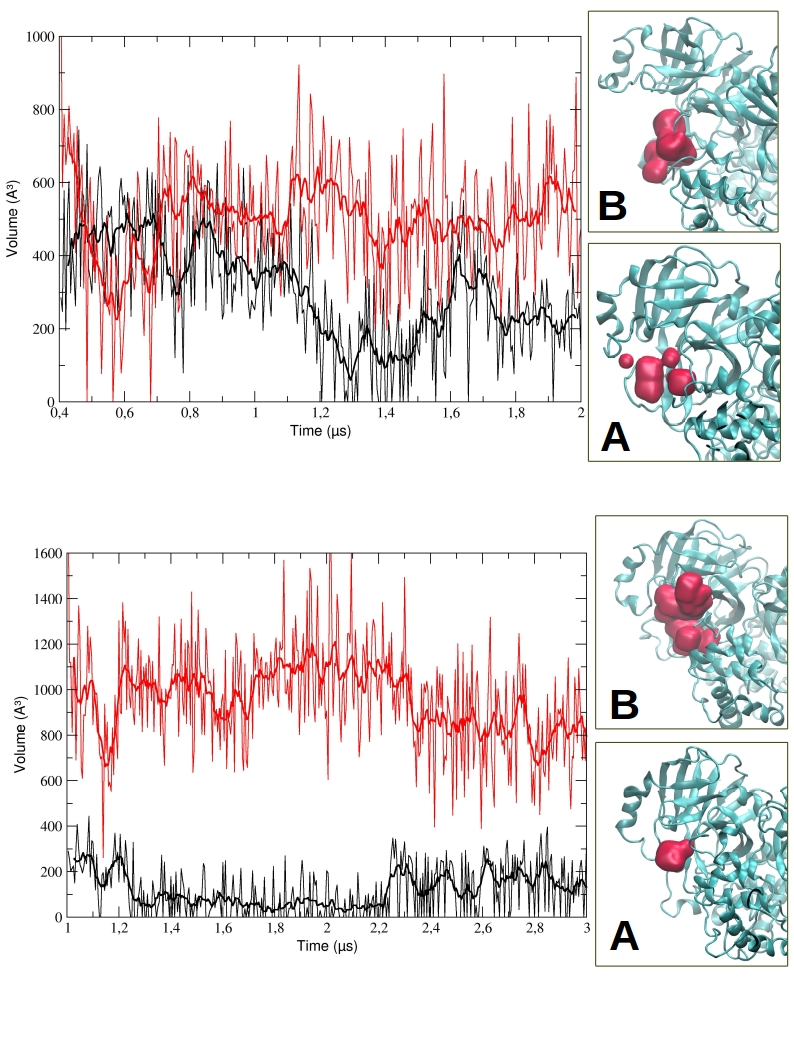

Supplement: Supplementary file 1 [file ijms-21-05346-s001.zip › SI_REsubmission_v2.docx]
